# Water deprivation compromises maternal physiology and reproductive success in a cold and wet adapted snake *Vipera berus*

**DOI:** 10.1093/conphys/coab071

**Published:** 2021-09-03

**Authors:** Mathias Dezetter, Jean François Le Galliard, Gaëtan Guiller, Michaël Guillon, Mathieu Leroux-Coyau, Sandrine Meylan, François Brischoux, Fréderic Angelier, Olivier Lourdais

**Affiliations:** 1 Sorbonne University, CNRS, IRD, INRA, Institut d’Écologie et des Sciences de l’Environnement (iEES Paris), 4 Place Jussieu, 75252 Paris Cedex 5, France; 2 Centre d’Études Biologiques de Chizé CNRS, UMR 7372, 79360 Villiers en Bois, France; 3 Ecole Normale Supérieure, PSL University, Département de Biologie, CNRS, UMS 3194, Centre de Recherche en Écologie Expérimentale et Prédictive (CEREEP-Ecotron IleDeFrance), 11 Chemin de Busseau, 77140 Saint-Pierre lès-Nemours, France; 4 Le Grand Momesson, 44130 Bouvron, France; 5 INSPE de Paris, Université Sorbonne, 10 rue Molitor, 75016 Paris, France; 6 School of Life Sciences, Arizona State University, Tempe, AZ 85287-4501, USA

**Keywords:** Dehydration, fecundity, reproduction, trade-off, water deprivation

## Abstract

Droughts are becoming more intense and frequent with climate change. These extreme weather events can lead to mass mortality and reproduction failure, and therefore cause population declines. Understanding how the reproductive physiology of organisms is affected by water shortages will help clarify whether females can adjust their reproductive strategy to dry conditions or may fail to reproduce and survive. In this study, we investigated the consequences of a short period of water deprivation (2 weeks) during early pregnancy on the physiology and behaviour of a cold- and wet-adapted ectotherm (*Vipera berus*). We also examined water allocation to developing embryos and embryonic survival. Water-deprived females exhibited significant dehydration, physiological stress and loss of muscle mass. These effects of water deprivation on water balance and muscle loss were correlated with the number of developing embryos. While water-deprived females maintained water transfer to embryos at the expense of their own maintenance, water deprivation also led to embryonic mortality. Overall, water deprivation amplifies the reproductive costs of water allocation to support embryonic development. The deleterious impacts of water deprivation on female current reproductive performance and on potential survival and future reproduction could lead to severe population declines in this species.

## Lay summary

We experimentally investigated the consequences of a short water deprivation during pregnancy in a cold- and wet-adapted boreal ectotherm. We show that water deprivation altered both maternal physiology and reproductive success, providing evidence that drought-induced dehydration and stress may lead to population declines in boreal ectotherms.

## Introduction

Understanding the effects of water shortage on the conservation status of terrestrial and semi-terrestrial animals is crucial since droughts are becoming more frequent and intense in some parts of the world with climate change (Cook *et al.*, 2014; Trenberth *et al.*, 2014). Droughts have direct physiological effects on organisms, such as an increased risk of lethal dehydration that can cause mass mortality events and rapid population declines in birds, mammals and amphibians (Albright *et al.*, 2017; McMenamin *et al.*, 2008; Tsuji *et al.*, 2018) with potential impacts on trophic networks and communities (DuBose *et al.*, 2019; McCreedy and Riper, 2015). However, the sub-lethal effects of water shortage on the physiology and life history of terrestrial animals remain relatively underexplored relative to the indirect effects mediated by trophic interactions (Amorim *et al.*, 2015; Sperry and Weatherhead, 2008). Documenting and quantifying physiological effects of water shortage will help us to understand and predict population responses to recurrent droughts in wild animals.

One sensitive and crucial life history stage that droughts can affect is reproduction. Indeed, droughts can alter reproduction through indirect effects involving changes in species interactions and habitat features (del Cacho *et al.*, 2013; McCreedy and Riper, 2015; McMenamin *et al.*, 2008), most notably through bottom-up effects on the food available to females before and during the reproductive period (Amorim *et al.*, 2015; Smith *et al.*, 2019; Sperry and Weatherhead, 2008). In addition, a very limited number of studies suggest that individual dehydration and physiological stress caused by droughts can have direct negative effects on reproductive performance in mammals, birds and reptiles independently from food availability (Nelson, 1993; Zani and Stein, 2018). For example, income-breeding lizards exposed to drought conditions have a lower body condition and reproductive success, which may cause recruitment failures even in the absence of food shortage (Cruz-McDonnell and Wolf, 2016; Zani and Stein, 2018). However, the extent to which such effects on reproduction are common or rare and the extent to which reproductive females can mitigate these effects remains largely unknown.

When water availability is limited during reproduction, a conflict exists between water allocation to offspring growth and development versus the allocation of water to the self-maintenance of the mother, which is similar to the well-known parent–offspring conflict for nutrients and energy (Crespi and Semeniuk, 2004; Kölliker *et al.*, 2015). This allocation conflict should translate into a water-based fecundity trade-off, where females producing more or larger offspring should face greater water demands during gravidity or pregnancy and also during post-natal growth in species with parental care that involves a stronger water expenditure (e.g. due to direct water provisioning to the offspring or increased water loss; Degen *et al.*, 2004; Tieleman *et al.*, 2003). Despite this allocation conflict, maintenance of homeostasis in situations of low water availability during reproduction might still be possible because females may cope with drought conditions through a variety of physiological and behavioural compensatory mechanisms (Ricklefs and Wikelski, 2002; Wingfield *et al.*, 2011). However, drought should be especially challenging for many viviparous species given the water needs of embryos *in utero* during late gestation and the longer length of the gestation period relative to oviparous species with precocial young (Furness *et al.*, 2015). Quantifying drivers and consequences of water-based reproductive trade-offs will help clarify whether females can compensate and adjust their reproductive strategy in response to drought conditions or may fail to reproduce and survive (Cruz-McDonnell and Wolf, 2016; Martin and Mouton, 2020).

Terrestrial squamate reptiles can allocate considerable amounts of water to sustain offspring development *in utero* (Brusch *et al.*, 2018; Lourdais *et al.*, 2015, 2017). Their reproduction and demography can be directly influenced by drought and rainfall regimes in the wild (Capehart *et al.*, 2016; Marquis *et al.*, 2008; Winne *et al.*, 2010). Even in cold and mesic environments where water is generally a less limited resource, these species may be critically dependent upon permanent access to free standing water to sustain the hydric costs of reproduction. First, squamate reptiles from more mesic and colder environments generally have higher basal metabolic rates, a lower cutaneous resistance to evaporative water loss (Cox and Cox, 2015) and a lower resistance to desiccation (Hertz *et al.*, 1979; Sexton and Heatwole, 1968), which may expose them to a greater risk of dehydration when water shortage occurs (Guillon *et al.*, 2014; Lourdais *et al.*, 2013a). Second, squamate reptiles inhabiting boreal or high-altitude habitats often have a viviparous reproduction mode (Foucart *et al.*, 2018; Li *et al.*, 2009), which may increase their vulnerability to water shortages during reproduction (Dupoué *et al.*, 2020; Jara *et al.*, 2019; Pincheira-Donoso *et al.*, 2013).

The European adder (*Vipera berus*) is a widespread Euro-Siberian snake adapted to cold- and wet-adapted environments. This viviparous species is a capital breeder that invest large amounts of energy and water into reproduction, which is characterized by a long gestation time (2–4 months), a high reproductive effort (average relative clutch mass of 45% maternal body mass; see Madsen and Shine, 1993) and a low breeding frequency associated with a relatively high longevity (Bauwens and Claus, 2019a). Manipulative studies have already investigated the effects of water deprivation on the reproduction of a closely related viper species living in semi-arid and warm climates (asp viper, *Vipera aspis*; see Dupoué *et al.*, 2015, 2016; Stier *et al.*, 2017). However, this topic has not been studied so far in this cold- and wet-adapted viper. The European adders have higher evaporative water loss rates, select more humid microhabitats and thus are more vulnerable to water shortage than the congeneric asp viper (Guillon *et al.*, 2014; Lourdais *et al.*, 2013a). Within the European distribution of the species, drought frequency and severity is increasing, notably in Western European populations and during the summer season when the females are pregnant (Spinoni *et al.*, 2018). Investigating how water shortages affect European adders during reproduction is therefore crucial to predict how the already-declining and threatened populations of adders (Gardner, 2019) will react to further threats caused by climate change. Here, we used a laboratory experiment to simulate a relatively short period of water deprivation (15 days) at the onset of pregnancy and analyse effects on the physiology, behaviour and reproductive biology of female adders. We experimentally manipulated access to drinking water at early stages of pregnancy and further examined intra-individual changes in muscle width, physiological state and embryonic development. In line with our scenario of a water-based fecundity trade-off, we hypothesized that water restriction would produce negative effects on female physiology and reproduction, especially in more fecund females. Building from recent knowledge in other snake species, we specifically predicted that (i) water deprivation should result in female dehydration and thus increased plasma osmolality (Dupoué *et al.*, 2015), increased baseline plasma corticosterone levels, increased oxidative stress and higher body mass loss (Dupoué *et al.*, 2016; Stier *et al.*, 2017); (ii) water-deprived females might engage in protein catabolism (increased structural muscle loss) and maintain water transfer to the eggs in order to support the water requirements of their embryos during development (Brusch *et al.*, 2018); (iii) water-deprived females should lower their thermal preference to behaviourally reduce their evaporative water loss rates (Ladyman and Bradshaw, 2003); and (iv) the intensity of these physiological and behavioural responses to water deprivation should be positively correlated with the number of developing embryos, reflecting the additive water constraints of fecundity (Lourdais *et al.*, 2017).

## Material and methods

### Study species

The European adder (*V. berus*) is a small viviparous snake widely distributed within the Palearctic region (Ursenbacher *et al.*, 2006). Like most vipers, European adders rely on sporadic feeding events for energy intake and capital breeding to fuel reproduction. Females accumulate significant amounts of energy prior to breeding and breed intermittently (Bauwens and Claus, 2019a). In our study area located in western France, ovulation occurs in early June and parturition occurs from mid-August to late September depending on summer climate conditions. During pregnancy, food intake is often reduced or absent due to behavioural trade-offs with thermoregulation and physical constraints associated with the abdominal space needed for embryo development during gestation (Bauwens and Claus, 2019b). The energetic costs of reproduction can lead to female emaciation and substantial mortality rates (Bauwens and Claus, 2019a). In addition, this boreal species exhibits higher water loss rates and uses more humid microhabitats than congeneric species from semi-arid environments (Guillon *et al.*, 2014). It is thus more likely to be vulnerable to water shortage, especially during reproduction when females display high metabolic rates, actively bask to maintain elevated thermal preferences and generally have high evaporative water loss rates (Lourdais *et al.*, 2013a).

### Capture and housing conditions

In May 2019, we captured 43 female adders from neighbouring sites in western France within the Ille-et-Vilaine district, Brittany. We assessed the reproductive status of females in the field by direct visual inspection and palpation. Once the snakes were brought to the laboratory, we used high-resolution ultrasonography (Sonosite microMaxx, Inc., Bothell, WA, USA) to confirm the reproductive status. All reproductive females were then measured (snout-vent-length, SVL ±0.5 cm), weighed (body mass, BM ± 0.1 g) and housed in outdoor enclosures for 2 weeks (located at the Centre d’Etudes Biologiques de Chizé, Villiers-en-Bois, France) prior to laboratory manipulations. Each enclosure was equipped with an artificial shelter, water was provided ad libitum and the vegetation provided a mosaic of basking sites and shelters. During this period, we fed the vipers twice with laboratory mice. After 2 weeks in the enclosures, we transferred all individuals to laboratory cages (56 × 41 × 14 cm) where they were individually housed during 2 additional weeks to habituate to laboratory conditions before the experiment started. Room temperature was held constant at 17°C. Each cage was equipped with a 22-W heating strip at one end to create a thermal gradient from ca. 20°C on the cold side to ca. 40°C on the warm side. Heat was provided for 6 hours per day. All cages were equipped with an artificial shelter and water was provided ad libitum. Vipers were not given food during these 2 weeks and remained fasting throughout the experiment, similar to natural conditions during pregnancy (Bauwens and Claus, 2019b).

### Assessment of reproductive effort and pregnancy stage

Before the start of the manipulation, we used high-resolution ultrasonography to quantify the number of developing embryos for each female. Because we did not keep the females until parturition, we could not measure the total litter mass to account for offspring size and we rather used the number of developing embryos as a proxy of reproductive effort. At this early developmental stage, we were able to distinguish viable embryos from non-viable embryonic units by the presence of a clearly visible embryo in ultrasonography (black ovoid shape in the upper part of the embryonic unit). We estimated embryonic stage by measuring the size of embryos, evaluating the remaining vitellus and assessing embryonic traits, using reference tables (Hubert and Dufaure, 1968) available for a closely related species (*V. aspis*; see Lorioux *et al.*, 2013). Ovulation is associated with skin shedding that provides a reliable index for the onset of gestation (Lorioux *et al.*, 2013). We thus determined this ovulatory ecdysis date of each viper by checking individuals during the housing period prior to the start of the experiment. Together with ultrasonography, this allowed us to control for pre-experimental differences in reproductive effort and breeding phenology prior to the start of our laboratory experiment.

### Experimental treatment

Individuals were randomly assigned to the control (*n =* 22) or water-deprived (*n =* 21) hydric treatment. For the water-deprived group, drinking water was removed for 15 days, which is grossly similar to the mild, chronic summer droughts repeatedly occurring over the past decade in western France. At the end of the treatment period, water-deprived snakes were provided water ad libitum immediately after blood-sampling (see below). Water-deprived and control individuals did not differ in BM or SVL at the beginning of the experiment (linear models, *lm*, package *stats*; initial BM, Treatment: ${F}_{1,41}=0.1,P=0.78$; SVL, Treatment: ${F}_{1,41}=0.04,P=0.84$). The experimental treatment occurred during early pregnancy for all individuals (onset of treatment: 18.4 ± 8.2% of developmental time; end of treatment: 38.4 ± 8.2% of developmental time). The estimated embryonic stage at the onset of the experimental treatment was 20–21 (ovulated eggs with ovoid shape and distinguishable embryos) and it reached 36–37 at the end (embryos exhibit a typical spiral body shape; see Hubert and Dufaure, 1968). During the experiment, housing conditions remained the same as described above. After the end of the experimental procedure all individuals were fed and monitored. After 2 weeks they were released at their original capture site to minimize captivity duration and stress in this sensitive species (Gardner, 2019). Procedures were performed in accordance with laws relative to the capture, transport and experimental use of *V. berus* (DREAL, CERFA number 13616*01) and approved by an independent ethical committee (Apafis #2019061722517180_v3).

### Body mass and muscle condition

In the absence of food intake, BM changes provide a reliable estimator of hydration state in squamate reptiles (Dupoué *et al.*, 2014). We thus weighed all individuals every 4 days (BM ± 0.01 g) throughout the experiment. At the end of the treatment period, we assessed the mass recovery associated with the water intake by weighing all individuals the day after subsequent access to water ad libitum. Individuals were not fed before these measurements. In vipers, the energy allocation into reproduction can lead to significant emaciation with the mobilization of both fat reserves and muscle protein (Dupoué and Lourdais, 2014). During pregnancy, the loss of structural muscles is linked to fecundity-dependent resource allocation (Lorioux *et al.*, 2016; Lourdais *et al.*, 2013b). Water deprivation increases the mobilization of structural protein and the associated release of bound water, which represent ca. 75% of the mass of skeletal muscles (Brusch *et al.*, 2018). The width of the tail further provides an accurate estimate of muscle dimensions in snakes and is a proxy for changes in muscular mass induced (Lorioux *et al.*, 2016). We measured the width of the base of the tail using an electronic pressure-sensitive specimeter (Absolute Digimatic, Mitutoyo, Japan) at the position of the sixth subcaudal scale. Tail width (TW ± 0.01 mm) was repeatedly measured (in triplicate) right after each blood sampling (see below) and mean TW was calculated each time.

### Blood parameters

We collected a blood sample from each individual two days before the start and on the last day of the experimental treatment, where females were sampled in a random order. We immediately collected blood upon removing the female from her cage (mean blood sample duration 4.4 ± 2.0 min) in order to measure baseline corticosterone (CORT) levels (Romero and Wikelski, 2001). We recorded body temperature at the time of blood sampling using an infrared thermometer (Raytek Raynger MX2) at a standard distance and angle (300 mm and 45°, therefore covering a 6-mm area) with a constant emissivity coefficient (0.95). Body temperature was measured right before the blood samples when snakes were undisturbed within their thermogradient. This method gives close estimates of cloacal temperature measurements in vipers without the negative side effects of handling stress (Shine *et al.*, 2002). We collected blood samples (150 μl) through cardiocentesis using a 1-ml syringe and a heparinized 29-gauge needle. Immediately after sampling, we allocated a small amount of blood into two 10-μl micro-capillary tubes and centrifuged them. We measured haematocrit (Hct; length of red blood cells/length of total sample) in duplicate for each blood sample. Next, for each sample, we transferred the remaining blood into Eppendorf tubes (0.5 ml) and centrifuged them for 3 minutes at 2000 × g. Plasma was stored at −28°C until laboratory analyses. We measured plasma osmolality from 10 μl aliquots using a vapour pressure osmometer (± 3 mOsm kg^−1^; Vapro2, Elitech group). We determined plasma CORT concentrations (ng ml^−1^) following a well-established radioimmunoassay protocol (see Dupoué *et al.*, 2016 for details). Samples were run in three assays (intra-assay variation: 7.58%, inter-assay variation: 8.84%). We examined the oxidative status of females by measuring the concentration of reactive oxidative metabolites (ROMs) as an index of oxidative damage (Costantini, 2016) and antioxidant capacity (OXY) as an index of defence (Costantini, 2011). We used d-ROMs colorimetric kits (MC003, Diacron International, Italy) to evaluate the activity of organic hyperoxides and OXY-absorbent test kits (MC435, Diacron International Italy) to assess the non-enzymatic ability of diluted plasma samples (1:100) to neutralize an oxidant attack from hypochlorous acid. We determined intra-plate coefficients of variations in ROMs (1.2%) and OXY (2.7%) using a pooled plasma sample three times in each 96-well plate (1 plate for ROMs, 1 for OXY).

### Thermoregulation

To assess changes in thermoregulatory behaviour, we collected body temperature (*T_b_*) of the snakes once at the beginning and once at the end of the water deprivation period. All measurements were conducted during the daytime activity period (11:30–15:00), and we measured *T_b_* (in triplicate) 4 times, at 30-minute intervals, while the vipers had access to a 20–40°C thermal gradient within their cages. We collected surface *T_b_* with an infrared thermometer following the method described above.

### Embryonic water intake and survival

To determine the effect of the hydric treatment on embryonic development, water transfer to embryos, and on the survival of embryos, we monitored several embryonic traits using high-resolution ultrasonography (see Lorioux *et al.*, 2013). We performed ultrasonography on Days 0 and 13 and 1 week after the end of the water deprivation period. At each time point, we estimated embryonic stages using reference tables (Hubert and Dufaure, 1968). Vipers are lecithotrophic organisms (i.e. allocation of energy to the offspring by the mother is completed prior to ovulation, into the developing follicles); hence changes in embryonic unit volumes during pregnancy are mostly driven by embryonic water intake (Dupoué *et al.*, 2015; Stewart, 2013). To evaluate water transfer to the embryos, we thus monitored the volume of embryonic units following a previously established method (see Dupoué *et al.*, 2015). We collected ultrasonography images in the sagittal view of the most cranial and most caudal embryonic units. Then, we measured the height, length and bicone (form) of the embryonic unit and followed the method described in Maritz and Douglas (1994) to estimate embryonic unit volumes (cm^3^). We quantified embryonic mortality by counting living embryos at the onset and at the end of the treatment period. At the end of the treatment period, we could clearly distinguish living from dead embryos by the presence of a heartbeat and body movements.

### Statistical analyses

All statistical analyses were performed using R software version 3.6.3 (R Core Team, 2020). To investigate how viper morphology (BM, TW), physiology (plasma osmolality, haematocrit, baseline CORT, ROMs, OXY) and body temperature (*T_b_*) were affected by experimental conditions, we used univariate linear mixed effects models fitted with the *lme* function of the *nlme* package (Pinheiro *et al.*, 2019). For each univariate model, treatment group (control or water deprived), session (effect of time), females’ reproductive effort (number of living embryos at the onset of the treatment period) and their interaction were included as fixed factors. Individual identity was treated as a random effect. We dropped all non-significant interactions from these models using marginal *F* tests (*anova*, package *stats*). We further used pairwise post hoc Tukey tests (*emmeans*, package *emmeans*) to investigate statistical differences between treatment groups at each measurement time (session). We made sure that the residuals of our models did not significantly differ from a normal distribution (Shapiro–Wilk test, all *P* > 0.05) and ensured that all models met the assumptions of homoscedastic and normal residuals using diagnostic plots.

**Figure 1:**
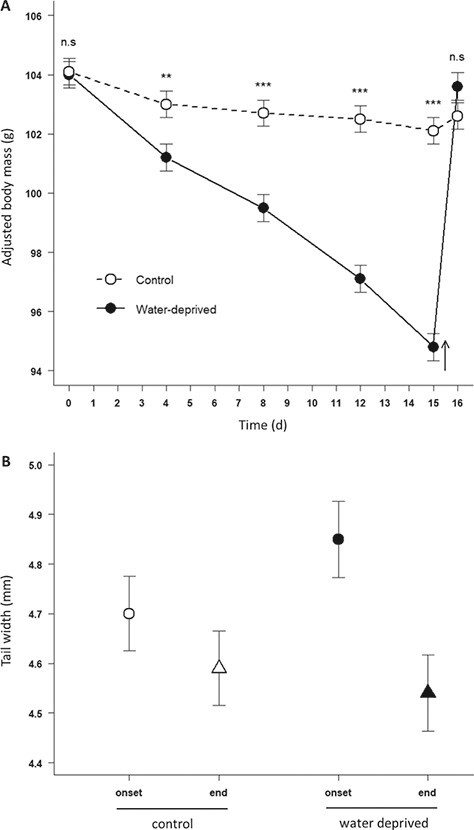
(**A**) Effect of the hydric treatment on body mass (mean ± SE; control individuals: open circles, dashed line; water-deprived individuals: filled circles, full lines). Body mass values were adjusted to initial body mass. Arrows indicate the end of the dehydration period, and subsequent access to water ad libitum for water-deprived females. Significant differences from post hoc tests between control and water-deprived females are symbolized: ^***^P < 0.001; ^**^P < 0.01; ^*^P < 0.05; n.s. nonsignificant. (**B**) Effect of water deprivation on changes in TW (mm) of pregnant female adders. Open dots represent means (± SE) for control females and filled dots represent means (± SE) for water-deprived females. Circles represent values at the onset of the experimental period and triangles represent values at the end.

To assess the effect of the experiment on the pattern of BM loss, BM was the dependent variable; initial BM was included as a linear covariate; and treatment, session and their interaction were included as fixed factors. To investigate how hydric treatment and reproductive effort were related to the water intake subsequent to the end of the water deprivation (ΔBM between Day 15 and Day 16; see Fig. 1A), we built a linear model (*lm*, package *stats*) with post-treatment water intake as the dependent variable; initial BM as a linear covariate; and hydric treatment, reproductive effort and their interaction as fixed factors. We removed one female (reproductive effort = 12 living embryos) from this analysis because she reached a reversible humane endpoint and had to be manually rehydrated. We added blood sample duration and body temperature as fixed factors when testing for the effects on baseline CORT. We also log-transformed CORT to meet the assumptions of homoscedastic and normal residuals.

To further investigate the correlations between physiological variables, we performed independent linear regressions between pairs of traits. First, we built linear regressions with post-treatment water intake as the dependent variable and physiological response traits as a fixed factor (either Δosmolality, ΔCORT or ΔROMs where Δ indicates the intra-individual change during the experiment). Second, we built linear regressions with either ΔCORT or ΔROMs as the dependent variable and Δosmolality as the fixed factor. Lastly, we built linear regressions between changes in TW and either ΔCORT, Δosmolality or ΔROMs as the fixed factor.

To determine the effects of water deprivation on embryonic development, we treated embryonic unit volume and embryonic stages as the dependent variables in two separate models, with hydric treatment, days since ovulation and their interaction as fixed factors. Embryonic unit position (i.e. cranial or caudal) and reproductive effort were treated as fixed factors when testing for the effects on embryonic unit volume. We further used a generalized linear model (*glm*, family *quasibinomial*, package *stats*) to investigate the effects of water deprivation on embryonic mortality with hydric treatment as a fixed factor. We used the family *quasibinomial* rather than *binomial* because our model was slightly over-dispersed (φ = 2.96). In order to determine if individual reproductive effort and physiological response traits (Δosmolality, ΔHct, ΔCORT, ΔROMs, ΔOXY) best predicted variation in embryonic mortality, we built six independent generalized linear models where each of these variables was included as a covariate. We next compared all models using a quasi-AIC (package *AICcmodavg*) model selection approach.

**Figure 2:**
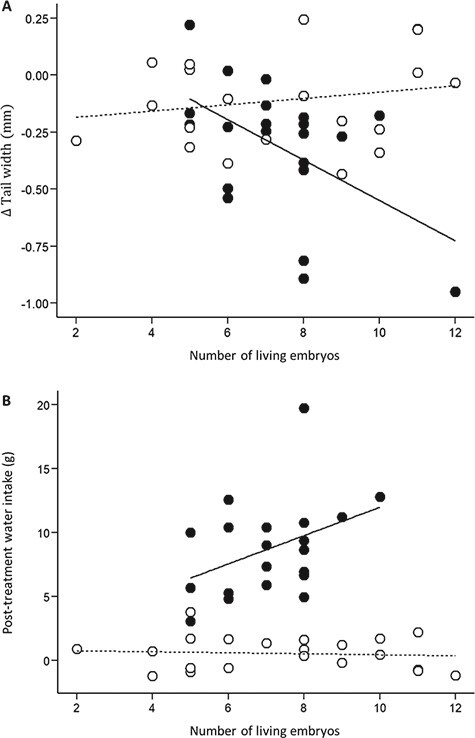
Influence of reproductive effort on (**A**) changes in TW (mm) during the experiment and on (**B**) water intake (g) following access to drinking water at the end of the experiment. Open circles are females from control group and filled circles are females from water-deprived group. Trend lines are included for significant relationships. One female (reproductive effort = 12 living embryos) was removed from the water intake analysis (B) because she had to be rehydrated manually.

## Results

### Maternal morphology

We observed a significant BM loss throughout the experiment (Session: ${F}_{\mathrm{3,123}}=226.8,P<0.001$) and BM loss was dependent on the initial BM (positive slope: ${F}_{1,39}=3927.0,P<0.001$) and hydric treatment (Treatment: ${F}_{1,39}=9.8,P=0.003$, Treatment × Session: ${F}_{\mathrm{3,123}}=87.4,P<0.001)$. Water deprivation induced a higher rate of BM loss in pregnant females (post hoc; $P<0.05;$  Fig. 1A). During the post-treatment water intake, water-deprived individuals abundantly drank water and rapidly gained mass to attain a BM that was similar on average to that of control snakes (post hoc test on last BM value; $P=0.188$; Fig. 1A). We also found that post-treatment water intake was dependent on the initial BM (${F}_{1,37}=6.2,P=0.02$) and the interaction between hydric treatment and reproductive effort (Treament × Reproductive effort: ${F}_{1,37}=5.4,P=0.02$). Within the water-deprived group, mass gain during the rehydration was positively related to the number of live embryos (Fig. 2A).

In addition, the TW of water-deprived females decreased more than in control individuals on average (Treatment × Session: ${F}_{1,39}=4.4,P=0.04$, Fig. 1B). The change in TW was also influenced by reproductive effort (Reproductive effort: ${F}_{1,39}=4.4,P=0.04$) and a three-way interaction between treatment, time and reproductive effort (Treatment × Session × Reproductive effort: ${F}_{1,39}=8.8,P=0.005$). The TW loss was positively related to the number of live embryos within the water-deprived group but not in the control group (Fig. 2B).

### Maternal physiology and thermal preferences

**Figure 3:**
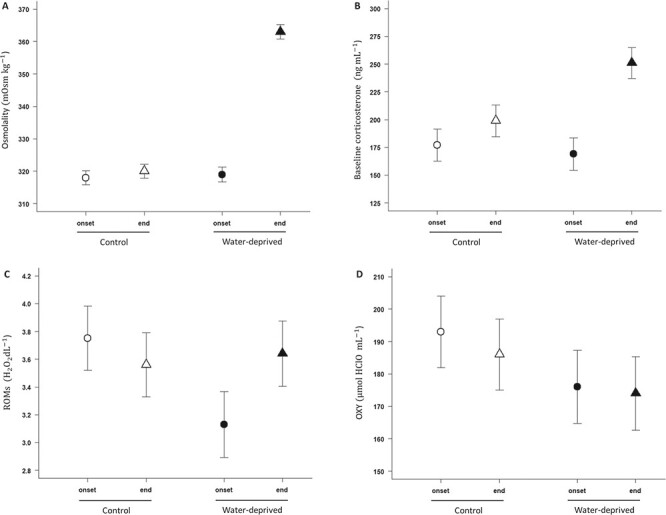
Effect of water deprivation on (**A**) plasma osmolality (mOsm kg^−1^), (**B**) corticosterone plasma concentrations (ng ml^−1^) and plasma (**C**) ROMs mg H_2_O_2_ dl^−1^) and (**D**) total antioxidant capacity (OXY, HClO ml^−1^). Open dots represent means (± SE) for control females and filled dots represent means (± SE) for water-deprived females. Circles represent values at the onset of the experimental period and triangles represent values at the end. (A) Plasma osmolality, (B) corticosterone plasma concentrations and (C) ROMs increased in the water-deprived group, whereas it did not change in the control group. (D) Total antioxidant capacity was not influenced by hydric treatment.

Plasma osmolality increased in water-deprived pregnant females throughout the experiment while there was no significant change in the control group (Treatment × Session: ${F}_{1,40}=145.3,P<0.001$; Table 1, Fig. 3A). On the contrary, haematocrit was not influenced by the hydric treatment (*F*_1,40_ = 0.003, *P* = 0.96), the session (*F*_1,41_ = 1.0, *P* = 0.32), their interaction (*F*_1,41_ = 1.3, *P* = 0.25) or reproductive effort (*F*_1,40_ = 0.4, *P* = 0.50). Baseline CORT was influenced by the interaction between treatment group and time (Treatment × Session: ${F}_{1,32}=5.3,P=0.03)$, increased slightly with the duration of the blood sample $({F}_{1,32}=4.4,P=0.04)$, and also increased with the body temperature during blood sampling $({F}_{1,32}=8.3,P=0.006)$. Baseline CORT levels did not differ between treatment groups at the onset of the manipulation (post hoc; $P=0.38$), but were slightly higher in the water-deprived group at the end of treatment (post hoc; $P=0.04$; Table 1; Fig. 3B).

**Table 1:** Summary statistics of the physiological parameters measured on pregnant European adders from control and water-deprived groups at the onset and at the end of water deprivation

		Hydric treatments		
		Control (*n* = 22)	Water deprived (*n* = 21)	
	Variables	Mean (± s.e.m.)	Mean (± s.e.m.)	Post hoc
Onset	Osmolality (mOsm.kg^−1^)	318 (2.21)	319 (2.26)	*P* = 0.77
	Haematocrit (%)	24.2 (0.82)	24.3 (0.84)	*P* = 0.96
	Baseline CORT (ng ml^−1^)	177 (14.50)	169 (14.70)	*P* = 0.38
	ROMs (H_2_O_2_ dL^−1^)	3.75 (0.23)	3.13 (0.24)	*P* = 0.07
	OXY (μmol HClO ml^−1^)	193 (11.0)	176 (11.3)	*P* = 0.28
	*T_b_* (°C)	31.6 (0.34)	30.8 (0.34)	*P* = 0.10
End	Osmolality (mOsm.kg^−1^)	320 (2.21)	363 (2.26)	*P* < 0.001
	Haematocrit (%)	23.5 (0.82)	24.7 (0.84)	*P* = 0.31
	Baseline CORT (ng ml^−1^)	199 (14.30)	251 (14.00)	*P* = 0.04
	ROMs (H_2_O_2_ dL^−1^)	3.56 (0.23)	3.64 (0.24)	*P* = 0.81
	OXY (μmol HClO ml^−1^)	186 (11.0)	174 (11.3)	*P* = 0.45
	*T_b_* (°C)	32.0 (0.34)	31.6 (0.35)	*P* = 0.44

Oxidative damage (ROMs) was also significantly influenced by the interaction between hydric treatment and time (Treatment × Session: ${F}_{1,40}=5.7,P=0.02)$. Plasma ROMs increased in the water-deprived group throughout the experiment, whereas plasma ROMs of the control group did not change (Table 1; Fig. 3C). In contrast, the non-enzymatic antioxidant capacity remained relatively constant during the study (plasma OXY; Table 1; Fig. 3D). The plasma OXY was not influenced by the hydric treatment (*F*_1,39_ = 1.7, *P* = 0.21), the session (*F*_1,40_ = 0.3, *P* = 0.60), their interaction (*F*_1,40_ = 0.1, *P* = 0.77) or reproductive effort (*F*_1,39_ = 0.5, *P* = 0.48). We also found no effect of water deprivation on female *T_b_* (Treatment × Session: ${F}_{\mathrm{1,251}}=0.1,P=0.72$; Table 1).

Post-treatment water intake was positively correlated with intra-individual changes in plasma osmolality (*F*_1,40_ = 181.7, *P*$<0.001$) and in ROMs (*F*_1,40_ = 33.5, *P* = 0.007), but not with changes in baseline CORT (*F*_1,33_ = 1.2, *P* = 0.28). ΔROMs was positively correlated with Δosmolality (*F*_1,41_ = 4.9, *P* = 0.03) but not with ΔCORT (*F*_1,34_ = 1.7, *P* = 0.19). TW loss during the treatment period was positively correlated with both Δosmolality (*F*_1,41_ = 11.6, *P* = 0.001) and ΔCORT (*F*_1,34_ = 6.9, *P* = 0.01), but not with ΔROMs (*F*_1,41_ = 1.6, *P* = 0.22) (see supplementary information).

### Effects on water transfer to the embryos and embryonic survival

Embryonic unit volume was not influenced by hydric treatment$({F}_{1,40}=0.0,P=0.86)$nor by the interaction between hydric treatment and number of days since ovulation $({F}_{\mathrm{1,198}}=0.9,P=0.34)$and by reproductive effort$({F}_{1,40}=1.3,P=0.25)$. However, embryonic unit volume was affected by embryonic unit position $({F}_{\mathrm{1,198}}=9.9,P=0.002)$and number of days since ovulation $({F}_{\mathrm{1,198}}=67.8,P<0.001)$. Volume increased with the number of days since ovulation, and embryonic units in a posterior position had greater volumes than embryonic units in an anterior position. The embryonic stage was also not affected by hydric treatment $({F}_{1,41}=2.6,P=0.11)$ or its interaction with the number days since ovulation $({F}_{\mathrm{1,193}}=3.1,P=0.07$), but it naturally increased with the number of days since ovulation $({F}_{\mathrm{1,193}}=319.2,P<0.001$).

We found a significant main effect of hydric treatment on embryonic mortality throughout the experimental period $(\mathrm{X}2=4.6,P=0.03)$. On average, water-deprived females lost 33.9% (± 6.2%) of their embryos, while control individuals lost 15.9% (± 6.1%) of their embryos. The best model explaining variation in embryonic mortality included ΔCORT as a fixed factor (see supplementary information): a stronger increase in baseline CORT throughout the experiment was related to a higher embryonic mortality $({\mathrm{X}}^2=6.9,P=0.008)$. When building a model with both hydric treatment and ΔCORT as fixed factors, the main treatment effect was no longer significant whereas the effect of ΔCORT remained significant (Treatment:${\mathrm{X}}^2=0.8,P=0.36,\Delta \mathrm{CORT}:{\mathrm{X}}^2=5.1,P=0.02)$.

## Discussion

Investigating how water shortage influences reproduction is crucial to predict how natural populations of terrestrial animals may respond to the increasing frequency and severity of droughts independently from changes in the habitat and in trophic interactions (Cruz-McDonnell and Wolf, 2016; Martin and Mouton, 2020). Our study shows that short-term water deprivation during pregnancy compromises maternal physiology (increased dehydration and glucocorticoids levels) and directly impairs reproductive performance (increased embryonic mortality) in a cold-adapted, viviparous ectotherm. In the water restricted group, females with more developing embryos faced a more pronounced dehydration and showed evidence of a more pronounced loss of muscle mass, providing strong evidence of increased water demands due to embryos during pregnancy. We hypothesize that the combined cost of water deprivation on female reproductive performance, as shown here, and possibly also on female survival, could lead to severe population declines in this species.

### Water deprivation increases the physiological costs of reproduction

The sharp increase in plasma osmolality during the 2 weeks experiment from normosmotic values (320 mOsm.kg^−1^) to hyperosmotic values in water-deprived females (360 mOsm.kg^−1^) and the fact that water-deprived females immediately drank when re-exposed to water, strongly suggest that female adders were significantly dehydrated (Dupoué *et al.*, 2015, 2014; Moeller *et al.*, 2013). Such a level of body dehydration can affect individual performance and lead to significant physiological and fitness costs (Lorenzon *et al.*, 1999; Moeller *et al.*, 2013). We found no effect of water deprivation on changes in haematocrit, which is consistent with a previous study in asp vipers (Dupoué *et al.*, 2015). One potential explanation is that haematocrit remained low despite severe dehydration to sustain the optimal blood fluidity needed for maternal-foetal O_2_ transfer during gestation.

In accordance with our initial predictions, water-deprived females exhibited a significant dehydration (increased plasma osmolality), some degree of physiological stress (increased baseline CORT and ROMs) and an increased body mass loss. They also exhibited greater loss of their tail musculature (assessed by a decrease in the TW) than control females. The increase in baseline CORT in water-deprived females was likely a classical stress response (Brusch *et al.*, 2020; Dupoué *et al.*, 2016) and might be involved in some downstream changes in energetic pathways, such as the higher protein catabolism and muscle wasting following chronic water restriction (Brusch *et al.*, 2018; Lorioux *et al.*, 2016; Lourdais *et al.*, 2013b). This scenario was further supported by the facts that changes in tail musculature were correlated with the increase in baseline CORT and in plasma osmolality. We also found that water deprivation led to an increase in plasma ROMs without significant changes in plasma antioxidant concentrations. The resulting imbalance between oxidative damage and antioxidant capacity is likely to further augment the physiological costs of reproduction (Metcalfe and Monaghan, 2013; Speakman and Garratt, 2014). The increase in ROMs was positively correlated with both post-treatment water intake and the increase in plasma osmolality.

This pattern adds to a growing number of studies showing that dehydration can alter maternal oxidative status. For example, female *V. aspis*, a congeneric south-European species, exhibit an oxidative shielding response when they are exposed to a water deprivation using a similar manipulative design (Stier *et al.*, 2017). In sharp contrast, female European adders were not able to protect themselves from oxidative damage, suggesting that mechanisms involved in the regulation of the oxidative balance are potentially more sensitive to water deprivation in this boreal species. Further, the severe BM loss, TW loss and physiological stress that we observed in water-deprived females is likely to indicate an altered individual condition and quality, which may reduce their future survival (Lorioux *et al.*, 2016; Lourdais *et al.*, 2013b). The loss of body condition and energetic reserves is also expected to alter future reproduction and can represent a substantial cost of reproduction for this capital breeder (Bauwens and Claus, 2019a; Winne *et al.*, 2006). Taken together, our results suggest that chronic water deprivation during early pregnancy may lead to severe physiological costs that are likely to negatively impact female survival and future reproduction. However, water-deprived snakes in natural conditions may behaviourally hydroregulate by selecting micro-habitats with a higher humidity, which could possibly buffer the detrimental effects of droughts (Guillon *et al.*, 2014; Pintor *et al.*, 2016; Rozen-Rechels *et al.*, 2020). These behavioural responses will depend on the availability of humid micro-habitats, which can be reduced by human activity and climate change (Gorissen *et al.*, 2017). Further conservation studies are required to investigate how European adders may compensate for drought-induced effects through hydroregulation and how habitats may act as a buffer against ecological consequences of climate change.

### Water deprivation alters reproductive performance

Previous studies in snakes ascribed the impact of drought on reproduction to indirect effects on food resources rather than to direct effects of water shortage *per se* (Rose and Todd, 2017; Smith *et al.*, 2019; Sperry and Weatherhead, 2008). Instead, our study demonstrates that water deprivation early in gestation can directly cause higher embryonic mortality. Similar conclusions were reached in recent studies where droughts led to reproductive failure in a rattlesnake and a lizard species (Capehart *et al.*, 2016; Zani and Stein, 2018). These studies hypothesized that water shortage decreased clutch sizes via resorption of follicles and increased abortion. Water is an important ‘resource’ to support embryonic development, notably for somatic growth, since water is critically needed to convert yolk reserves into embryonic tissues (Brace and Cheung, 2014; Lourdais *et al.*, 2015; Thompson and Speake, 2002). It is likely that water-deprived females in our study thus failed to meet the minimum water requirements of their developing embryos. In addition, our results suggest that a potential causal link between ΔCORT and embryonic mortality was underlying the main treatment effect. We note that embryonic water requirements are likely to be higher during late gestation since water demands of embryos increase exponentially following the pattern of somatic growth (Lourdais *et al.*, 2015, 2017). Therefore, we predict even stronger effects on reproductive failures when female adders face a severe drought during late gestation.

### Water-based fecundity tradeoff and parent-offspring conflict for water

Female fecundity was a significant predictor for two response traits. First, the water uptake during the rehydration phase was positively correlated with the number of developing embryos, suggesting that thirst was proportional to reproductive effort. Second, the loss of muscles of water-deprived females was positively correlated with the number of embryos, suggesting that muscle catabolism during water deprivation was also proportional to reproductive effort. This suggests greater responses to water restriction in more fecund females, which is consistent with previous findings of water-based reproductive trade-offs in vipers (Dupoué *et al.*, 2015; Lourdais *et al.*, 2015). Water availability therefore generates significant constraint on reproductive effort and optimal fecundity, similar to energy constraints caused by food restriction. Further, limited water availability should increase the conflict between maintenance of maternal water balance (linked with female survival and future reproduction) and allocation of water to developing embryos (linked with current reproductive event and offspring fitness) during reproduction in vipers (Dupoué *et al.*, 2015). Herein, we found that water-deprived females maintained water transfer to their developing embryos at a similar rate to that of control females because embryonic unit volume was not influenced by water restriction. One possibility is that water-deprived females were able to maintain water transfer into eggs and support the development of the surviving embryos because they stopped investing in embryos that died. In addition, we found a correlation between TW loss and the number of developing embryos, thus suggesting that muscle catabolism is a compensatory physiological response to support the water demand of the surviving embryos (Brusch *et al.*, 2018).

The capital breeder concept usually focuses on energetic resources, but can also be applied in terms of water resources, as female muscular reserves may provide an internal water resource to be invested into reproduction (Brusch *et al.*, 2018). It is therefore possible that selective pressures favour an increased muscle mass to support both energy and water demand for future reproduction. Our results suggest that despite embryonic mortality, females compromised their water balance and, therefore, their physiological health to support the demands of their surviving embryos (Brusch *et al.*, 2018; Dupoué *et al.*, 2015). Water deprivation could further influence the development of surviving embryos and ultimately impact the offspring phenotypes at birth and later in life (Brusch *et al.*, 2019; Dupoué *et al.*, 2016). In two viviparous squamate (*V. aspis* and *Zootoca vivipara*), Dupoué *et al.*, 2019 demonstrated that water deprivation can trigger some sex reversal mechanisms during the early embryonic development without affecting embryonic survival. Herein, we cannot elucidate if water-deprivation led to similar changes in the sex ratio of the litter or other characteristics of the offspring at birth. Further studies investigating transgenerational effects of water-deprivation during gestation and parent–offspring conflicts for water would strengthen our understanding of how drought can impact reproduction and offspring phenotypes. Water-deprived females did not lower their body temperature in order to reduce evaporative water loss rates and mitigate some of the consequences of water deprivation. On average, body temperatures (31–32°C, Table 1) were slightly below the preferred body temperature of female adders during pregnancy when measured independently from water availability (33–34°C in adult females, Herczeg *et al.*, 2007; Lourdais *et al.*, 2013a). Body temperatures were similar on average in control and water-deprived females, which suggest that body temperature was tightly regulated to meet the thermal requirements of embryos during early pregnancy independently from water availability (Lorioux *et al.*, 2013). In addition, maintaining elevated body temperatures despite water restriction can also prevent an extension of the pregnancy duration and likely induce greater physiological costs of reproduction for females.

### Implications for the conservation of European adders

Our laboratory manipulation indicates that the reproduction of the European adder, a boreal viper, is highly sensitive to water deprivation and, therefore, that droughts may lead to population declines in this species. In particular, droughts are likely to cause reduced recruitment (Capehart *et al.*, 2016; Zani and Stein, 2018) and may further induce long-lasting demographic effects on adult survival and future reproduction because of the physiological costs of water deprivation for pregnant females (Cruz-McDonnell and Wolf, 2016; Zani and Stein, 2018). Recruitment may not immediately rebound during years following droughts, especially since water deprivation can deplete muscular reserves in this capital breeder, and a higher post-breeding mortality may follow chronic summer droughts during gestation (Bauwens and Claus, 2019a). The effects of droughts on reproduction are likely to be stage dependent, where water deprivation in early stages (vitellogenesis) may lead to reduced investment into reproduction (i.e. resorptions of follicles; Capehart *et al.*, 2016; Zani and Stein, 2018) while in later stages (pregnancy) it may lead to greater physiological costs for the females (Lourdais *et al.*, 2015, 2017) and to embryonic mortality. These ecological consequences are likely to be even stronger if the demographic costs of water shortage are higher for larger females with more embryos. Independently of changes in food availability or their habitat, European adder populations are therefore likely to experience drought-induced declines.

Physiological and reproductive consequences of our laboratory-based short water deprivation are possibly exacerbated by captivity and handling stress. At the same time, we simulated a short period of water deprivation whereas it is predicted that droughts can be more intense in the wild spanning periods ranging from days to months (Cook *et al.*, 2014; Trenberth *et al.*, 2014). Intense and long droughts in natural populations are likely to have greater impacts on female physiology and reproduction than in our laboratory study. By the end of 21st century, both western Europe and northern Scandinavia are predicted to face an outstanding increase in drought frequency and severity, notably from spring to late summer when adders reproduce (Spinoni *et al.*, 2018). The species may therefore face population declines not only in the southern margins of its European distribution where the climate is warming rapidly, but also within its northern distribution in Scandinavia and Western Europe where droughts will become more frequent. This is likely to challenge the capacity of the species to track the predicted northward shift of its range distribution in warmer climates (Hickling *et al.*, 2005). In order to anticipate such population declines, our results suggest that corticosterone and plasma osmolality assays might offer reliable biological indicators of the hydric status of vipers and could therefore be useful to assess population vulnerability. In addition, management measures that focus on the conservation of wetlands and humid microhabitats may help mitigate the effects of droughts on European adder populations.

## Funding

This work was supported by the Agence Nationale de la Recherche under the ‘Aquatherm’ project (ANR-17-CE02-0013 to J.-F.L.G. and O.L.), Region Nouvelle-Aquitaine under the ‘Aquastress’ project (2018-1R20214 to O.L.) and the Departmental Council of Gironde under the ‘Climate Sentinels’ project (coordinators: Cistude Nature, Fanny Mallard). M.D. is supported by a doctoral grant from ED 227 Sorbonne University.

## Authors’ contributions

M.D., O.L. and J.-F.L.G. conceived the ideas and designed methodology. M.D., O.L, G.G. and M.G. captured snakes, contributed to experimental procedures and collected the data. F.A., S.M., M.L.-C. and F.B. led the laboratory analysis on blood samples and contributed in result interpretation. M.D. led the data analyses together with O.L. and J.-F.L.G. M.D. led the writing of the manuscript. All authors contributed critically in result interpretation and manuscript writing and gave final approval for publication.

## Conflict of interest

The authors declare no competing or financial interests.

## Data accessibility

Data will be deposited in Zenodo after acceptance.

## Supplementary Material

Dezetter_et_al_Supplementary_File_coab071
