# Supplementary material for: Water deprivation compromises maternal physiology and reproductive success in a cold and wet adapted snake Vipera berus
Source: Conserv Physiol. 2021 Sep 3;9(1):coab071. doi: 10.1093/conphys/coab071 (PMC8415537; doi:10.1093/conphys/coab071)

**SUPPLEMENTARY INFORMATION**

**Table S1.** Best statistical models describing effects of water deprivation treatment on maternal body mass and tail width. Individual identity was treated as a random factor and variance components were calculated for inter-individual ($\sigma_{ind}^{2}$) and intra-individual variation ($\sigma_{resid}^{2}$).

|  |  |  |  |  | **Variance components** | |
| --- | --- | --- | --- | --- | --- | --- |
| **Variable** | **Factor** | **df** | **F** | **P** | $\sigma_{ind}^{2}$ | $\sigma_{resid}^{2}$ |
| Body mass | **Initial body mass** | 1,39 | 3926.98 | **˂0.001** | 1.71 | 0.85 |
|  | **Treatment** | 1,39 | 9.83 | **0.003** |  |  |
|  | **Time** | 3,123 | 226.82 | **˂0.001** |  |  |
|  | **Fecundity** | 1,39 | 4.37 | **0.04** |  |  |
|  | **Treatment × Time** | 3,123 | 87.43 | **˂0.001** |  |  |
|  |  |  |  |  |  |  |
| Tail width | Treatment | 1,39 | 0.09 | 0.76 | 0.31 | 0.16 |
|  | Time | 1,39 | 2.36 | 0.13 |  |  |
|  | **Fecundity** | 1,39 | 4.43 | **0.04** |  |  |
|  | **Treatment × Time** | 1,39 | 4.40 | **0.04** |  |  |
|  | Treatment × Fecundity | 1,39 | 0.49 | 0.48 |  |  |
|  | Time × Fecundity | 1,39 | 0.44 | 0.44 |  |  |
|  | **Treatment × Time × Fecundity** | 1,39 | 8.77 | **0.005** |  |  |
|  |  |  |  |  |  |  |

**Table S2.** Best statistical model describing effects of water deprivation treatment on post-treatment water intake.

| **Variable** | **Factor** | **df** | **F** | **P** |
| --- | --- | --- | --- | --- |
| Post-treatment water intake | **Initial body mass** | 1 | 6.16 | **0.01** |
|  | Treatment | 1 | 0.06 | 0.80 |
|  | Fecundity | 1 | 2.73 | 0.11 |
|  | **Treatment × Fecundity** | 1 | 5.39 | **0.02** |
|  |  |  |  |  |

**Table S3.** Best statistical models describing effects of water deprivation treatment on maternal physiology. Individual identity was treated as a random factor and variance components were calculated for inter-individual ($\sigma_{ind}^{2}$) and intra-individual variation ($\sigma_{resid}^{2}$).

|  |  |  |  |  | **Variance components** | |
| --- | --- | --- | --- | --- | --- | --- |
| **Variables** | **Factor** | **df** | **F** | **P** | $\sigma_{ind}^{2}$ | $\sigma_{resid}^{2}$ |
| Osmolality | **Treatment** | 1,39 | 8.09 | **0.007** | 6.47 | 8.09 |
|  | Time | 1,41 | 1.13 | 0.29 |  |  |
|  | Fecundity | 1,39 | 1.52 | 0.22 |  |  |
|  | **Treatment × Time** | 1,41 | 145.57 | **˂0.001** |  |  |
|  | **Treatment × Fecundity** | 1,39 | 9.55 | **0.004** |  |  |
|  |  |  |  |  |  |  |
|  |  |  |  |  |  |  |
| Hematocrit | Treatment | 1,40 | 0.002 | 0.96 | 3.08 | 2.29 |
|  | Time | 1,41 | 1.02 | 0.32 |  |  |
|  | Fecundity | 1,40 | 0.47 | 0.49 |  |  |
|  | Treatment × Time | 1,41 | 1.33 | 0.25 |  |  |
|  |  |  |  |  |  |  |
|  |  |  |  |  |  |  |
| Log(CORT) | Treatment | 1,39 | 0.78 | 0.38 | 0.15 | 0.30 |
|  | Time | 1,32 | 1.07 | 0.31 |  |  |
|  | Fecundity | 1,39 | 1.58 | 0.21 |  |  |
|  | **Blood sample duration** | 1,32 | 4.41 | **0.04** |  |  |
|  | **Blood sample *T_b_*** | 1,32 | 8.33 | **0.007** |  |  |
|  | **Treatment × Time** | 1,32 | 5.26 | **0.03** |  |  |
|  |  |  |  |  |  |  |
|  |  |  |  |  |  |  |
| ROMs | Treatment | 1,40 | 3.55 | 0.06 | 0.89 | 0.61 |
|  | Time | 1,41 | 1.15 | 0.29 |  |  |
|  | Fecundity | 1,40 | 2.11 | 0.15 |  |  |
|  | **Treatment × Time** | 1,41 | 7.03 | **0.01** |  |  |
|  |  |  |  |  |  |  |
|  |  |  |  |  |  |  |
| OXY | Treatment | 1,40 | 1.19 | 0.28 | 26.52 | 44.26 |
|  | Time | 1,41 | 0.28 | 0.60 |  |  |
|  | Fecundity | 1,40 | 0.34 | 0.56 |  |  |
|  | Treatment × Time | 1,41 | 0.08 | 0.78 |  |  |
|  |  |  |  |  |  |  |
|  |  |  |  |  |  |  |
| *T_b_* | Treatment | 1,36 | 2.86 | 0.09 | 1.10 | 1.98 |
|  | Time | 1,255 | 1.65 | 0.20 |  |  |
|  | **Fecundity** | 1,255 | 6.05 | **0.01** |  |  |
|  | Treatment × Time | 1,255 | 0.97 | 0.32 |  |  |
|  |  |  |  |  |  |  |

List of acronyms: CORT = baseline plasma corticosterone concentration; ROMs = concentration of reactive oxidative metabolites; OXY = antioxidant capacity; Tb = body temperature

**Table S4.** Statistical models used to examine the effects of water deprivation treatment on embryonic development. Individual identity was treated as a random factor and variance components were calculated for inter-individual ( $\sigma_{ind}^{2}$) and intra-individual variation ($\sigma_{resid}^{2}$).

|  |  |  |  |  | **Variance components** | |
| --- | --- | --- | --- | --- | --- | --- |
| **Variables** | **Factor** | **df** | **F** | **P** | $\sigma_{ind}^{2}$ | $\sigma_{resid}^{2}$ |
| Embryonic stage | Treatment | 1,41 | 2.58 | 0.11 | 3.03 | 4.80 |
|  | **Time since ovulation** | 1,193 | 319.25 | **˂0.001** |  |  |
|  | Treatment × Time since ovulation | 1,193 | 3.15 | 0.07 |  |  |
|  |  |  |  |  |  |  |
|  |  |  |  |  |  |  |
| Embryonic unit volume | Treatment | 1,40 | 0.03 | 0.87 | 0.80 | 1.14 |
|  | **Time since ovulation** | 1,198 | 67.85 | **˂0.001** |  |  |
|  | Fecundity | 1,40 | 1.35 | 0.25 |  |  |
|  | **Embryonic unit position** | 1,198 | 9.88 | **0.002** |  |  |
|  | Treatment × Time since ovulation | 1,198 | 0.91 | 0.34 |  |  |
|  |  |  |  |  |  |  |
|  |  |  |  |  |  |  |

**Table S5.** Statistical models used to investigate the correlation between intra-change in different variables during the treatment period

| **Variables** | **Factor** | **df** | **F** | **P** | **R²** |
| --- | --- | --- | --- | --- | --- |
| Post-treatment water intake | Intercept | 1 | 0.16 | 0.69 |  |
|  | **Δ osmolality** | 1 | 181.71 | **< 0.001** | **0.82** |
|  |  |  |  |  |  |
| Post-treatment water intake | Intercept | 1 | 14.80 | < 0.001 |  |
|  | Δ CORT | 1 | 1.19 | 0.28 | 0.03 |
|  |  |  |  |  |  |
| Post-treatment water intake | Intercept | 1 | 33.47 | < 0.001 |  |
|  | **Δ ROMs** | 1 | 7.83 | **0.007** | **0.16** |
|  |  |  |  |  |  |
| Δ CORT | Intercept | 1 | 2.43 | 0.13 |  |
|  | Δ osmolality | 1 | 1.75 | 0.19 | 0.11 |
|  |  |  |  |  |  |
| Δ ROMs | Intercept | 1 | 0.60 | 0.44 |  |
|  | **Δ osmolality** | 1 | 4.88 | **0.03** | **0.11** |
|  |  |  |  |  |  |
| Δ Tail width | Intercept | 1 | 11.86 | 0.001 |  |
|  | **Δ CORT** | 1 | 6.96 | **0.01** | **0.17** |
|  |  |  |  |  |  |
| Δ Tail width | Intercept | 1 | 3.25 | 0.07 |  |
|  | **Δ osmolality** | 1 | 11.56 | **0.001** | **0.22** |
|  |  |  |  |  |  |
| Δ Tail width | Intercept | 1 | 24.78 | < 0.001 |  |
|  | Δ ROMs | 1 | 1.56 | 0.22 | 0.04 |

List of acronyms: CORT = baseline plasma corticosterone concentration; ROMs = concentration of reactive oxidative metabolites

**Table S6.** Statistical models used to investigate if intra-individual changes in physiological variables or fecundity influenced embryonic mortality.

| **Model number** | **Factor** | **df** | **X^2^** | **P** |
| --- | --- | --- | --- | --- |
| 1 | **Treatment** | 1 | 4.63 | **0.03** |
|  |  |  |  |  |
| 2 | **Δ CORT** | 1 | 6.93 | **0.008** |
|  |  |  |  |  |
| 3 | Treatment | 1 | 0.84 | 0.36 |
|  | **Δ CORT** | 1 | 5.14 | **0.02** |
|  |  |  |  |  |
| 4 | Fecundity | 1 | 0.002 | 0.96 |
|  |  |  |  |  |
| 5 | Δ osmolality | 1 | 0.13 | 0.72 |
|  |  |  |  |  |
| 6 | Δ Hematocrit | 1 | 0.10 | 0.74 |
|  |  |  |  |  |
| 7 | Δ ROMs | 1 | 2.25 | 0.13 |
|  |  |  |  |  |
| 8 | Δ OXY | 1 | 0.002 | 0.96 |

List of acronyms: CORT = baseline plasma corticosterone concentration; ROMs = concentration of reactive oxidative metabolites; OXY = antioxidant capacity

**Table S7.** QAICc-based model selection comparing the influence of fecundity and intra-individual changes in physiological variables on embryonic mortality.

| **Model** | **k** | **QAICc** | **Δ QAICc** | **wi** | **Q LogLik** |
| --- | --- | --- | --- | --- | --- |
| **Δ CORT** | 3 | **52.48** | 0.00 | 0.98 | - 22.85 |
| Δ ROMs | 3 | 61.93 | 9.45 | 0.01 | - 27.64 |
| Δ Osmolality | 3 | 63.92 | 11.44 | 0.00 | - 28.64 |
| Δ Hematocrit | 3 | 63.65 | 11.46 | 0.00 | - 28.65 |
| Fecundity | 3 | 64.05 | 11.57 | 0.00 | - 28.70 |
| Δ OXY | 3 | 64.05 | 11.57 | 0.00 | - 28.70 |

List of acronyms: CORT = baseline plasma corticosterone concentration; ROMs = concentration of reactive oxidative metabolites; OXY = antioxidant capacity

**Figure S1:** Ultrasound imaging at the beginning of the treatment period (developmental stage 20-21) of (A) a viable embryonic unit with a typical ovoid shape; (B) a clearly visible embryo (black circular shape within the embryonic unit); (C) unviable embryonic units with circular shape and no visible embryo; and (D) at the end of the treatment period (developmental stage 36-37) of a living embryo with a typical spiral body shape.


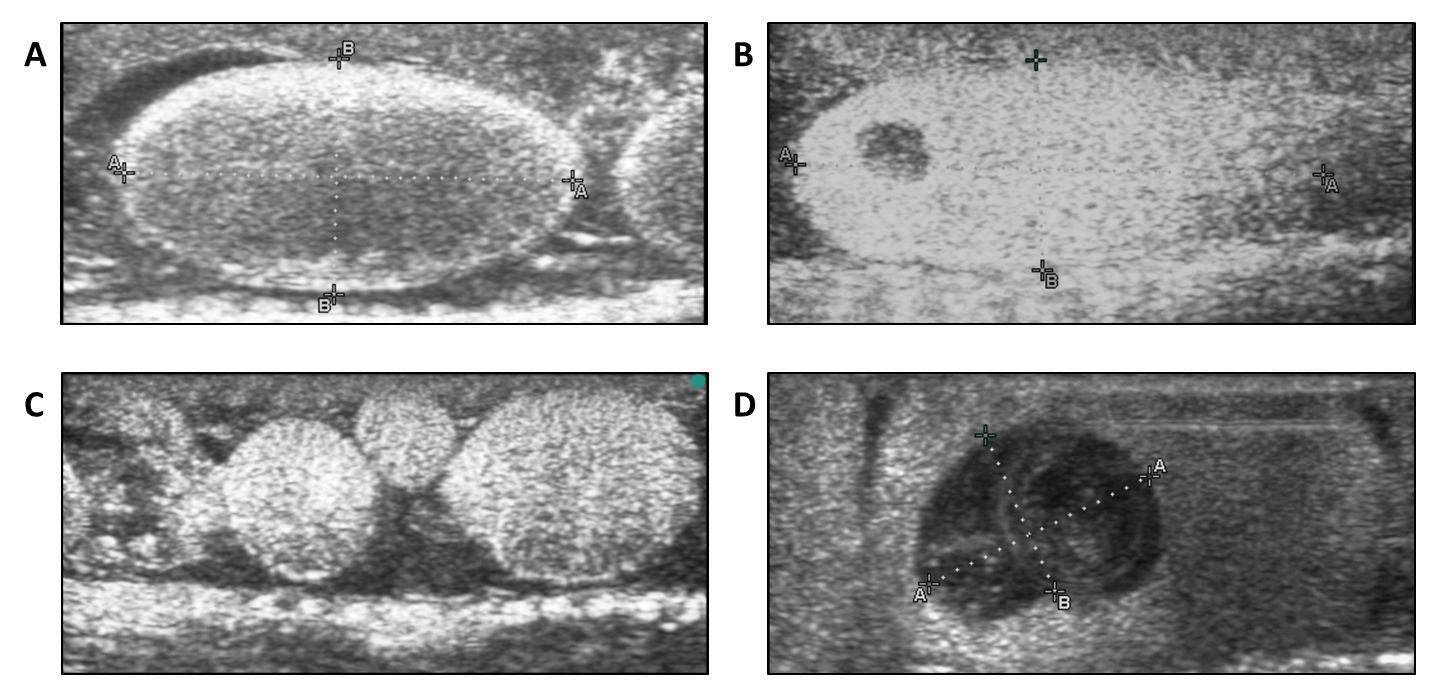


**Figure S2:** Correlation between (A) water intake (g) and intra-individual change in plasma osmolality (mOsm kg^-1^), and (B) between water intake (g) and intra-individual change in reactive oxygen metabolites (ROMs, mg H_2_O_2_ dL^-1^)


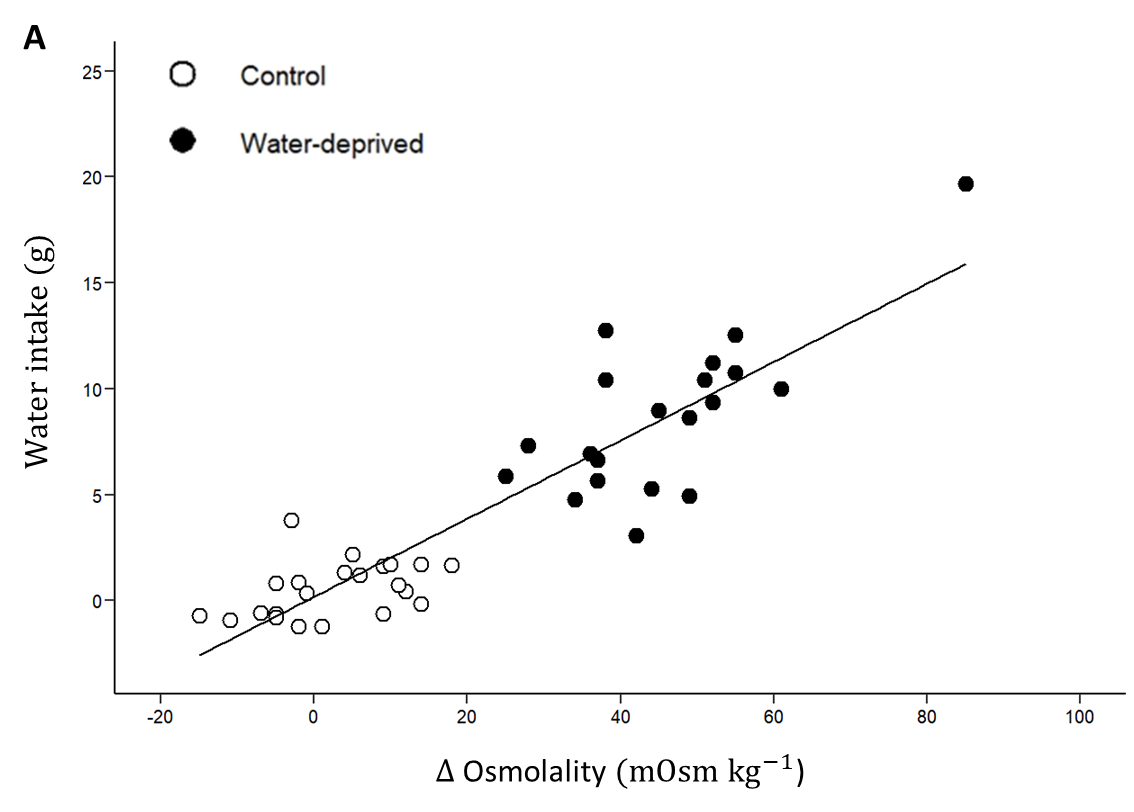

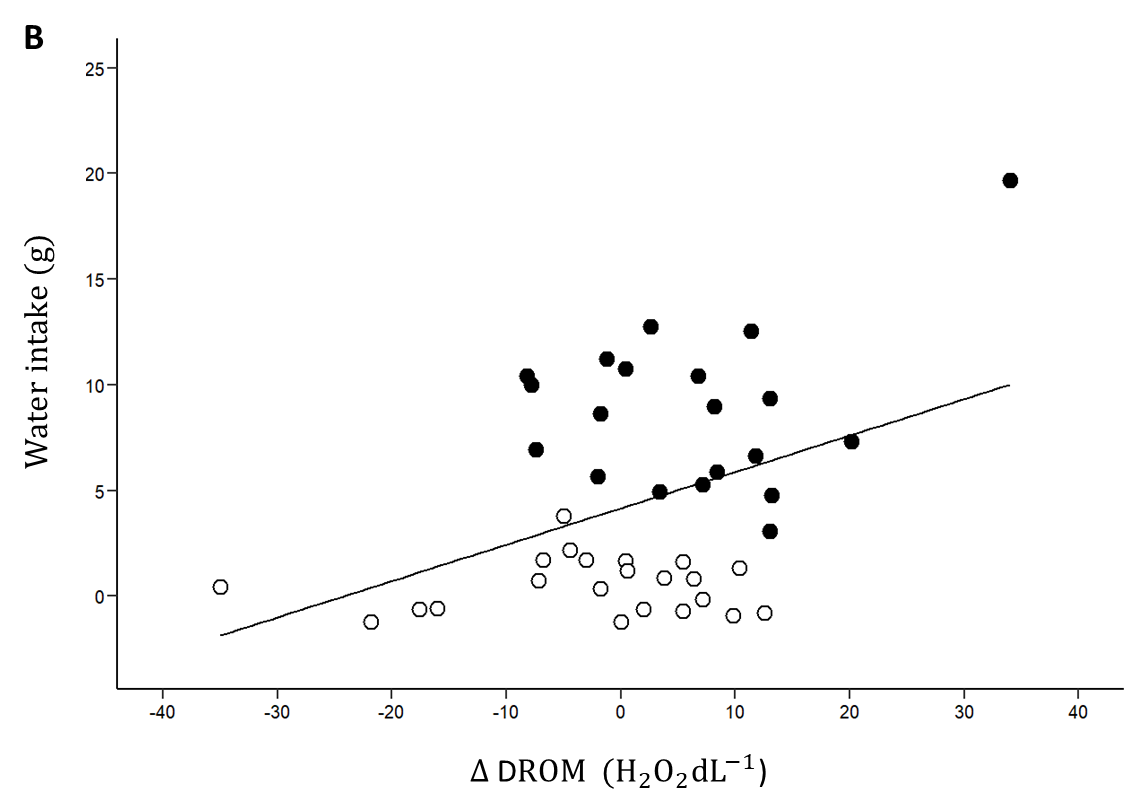


**Figure S3:** Correlation between intra-individual changes in reactive oxygen metabolites (ROMs, mg H_2_O_2_ dL^-1^) and in plasma osmolality (mOsm kg^-1^)


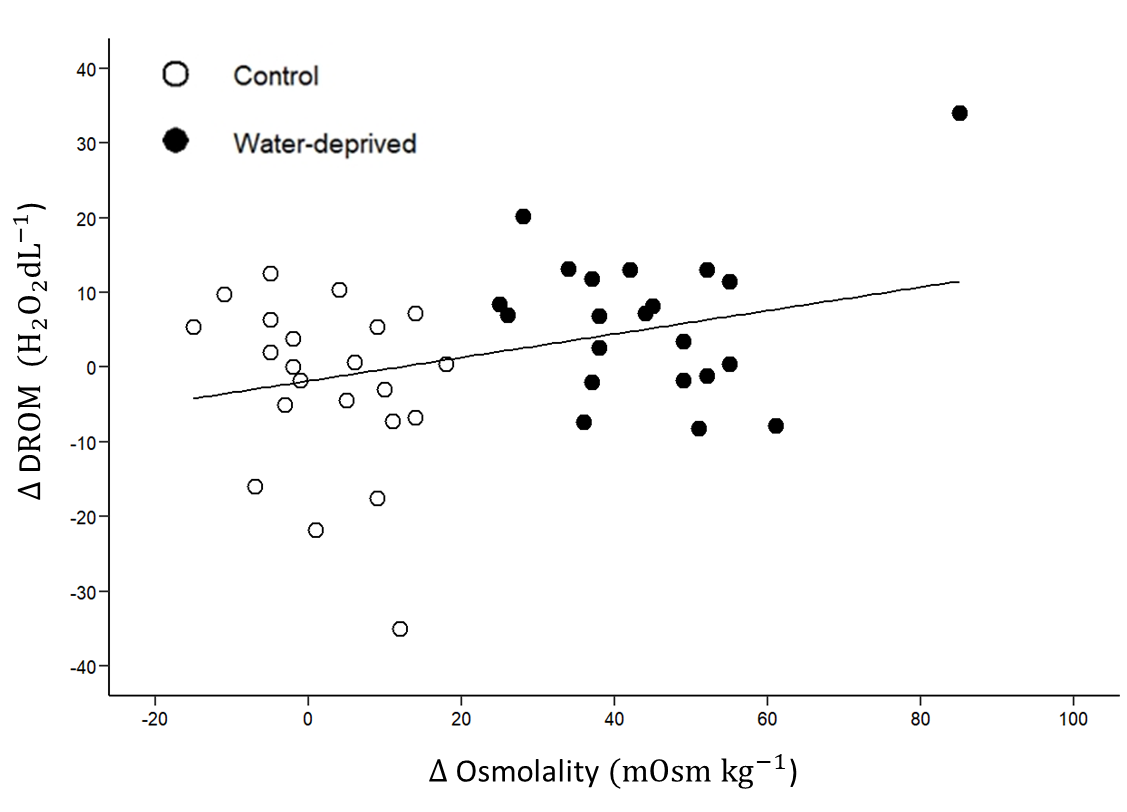


**Figure S4:** Correlation between (A) intra-individual changes in tail width (mm) and in plasma osmolality (mOsm kg^-1^) and (B) between intra-individual changes in tail width (mm) and in corticosterone plasma concentrations (ng mL^-1^)


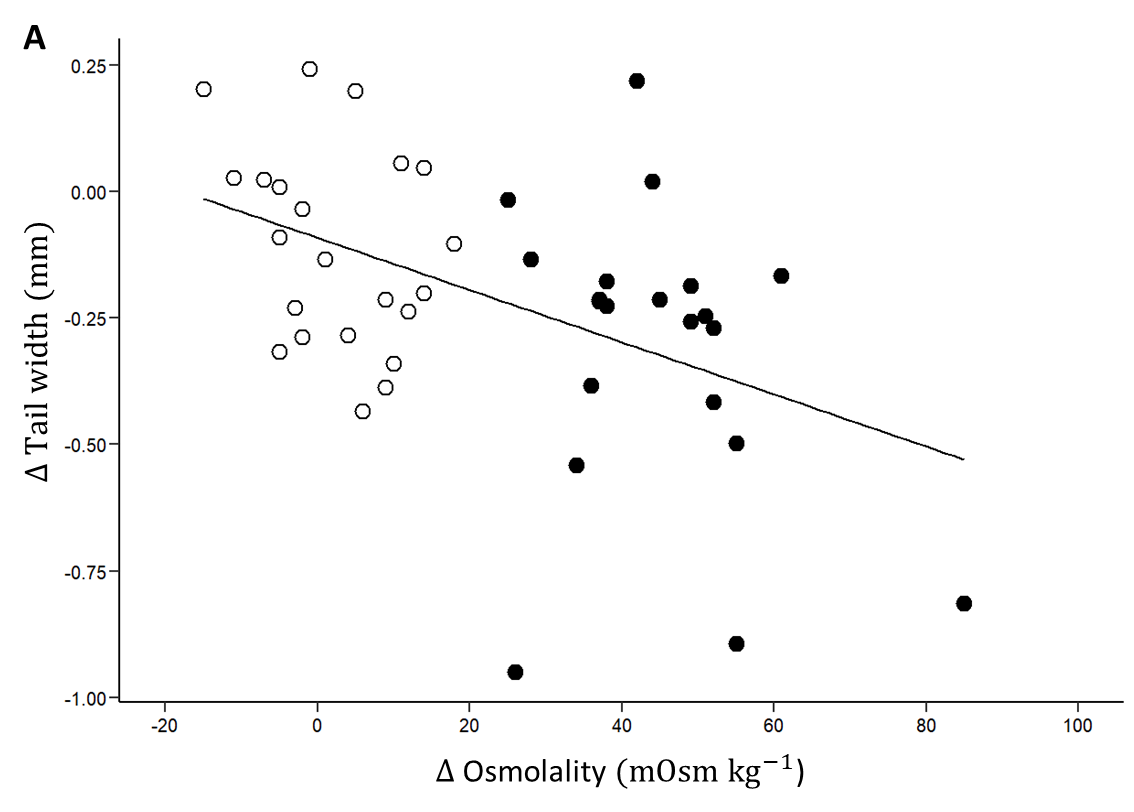

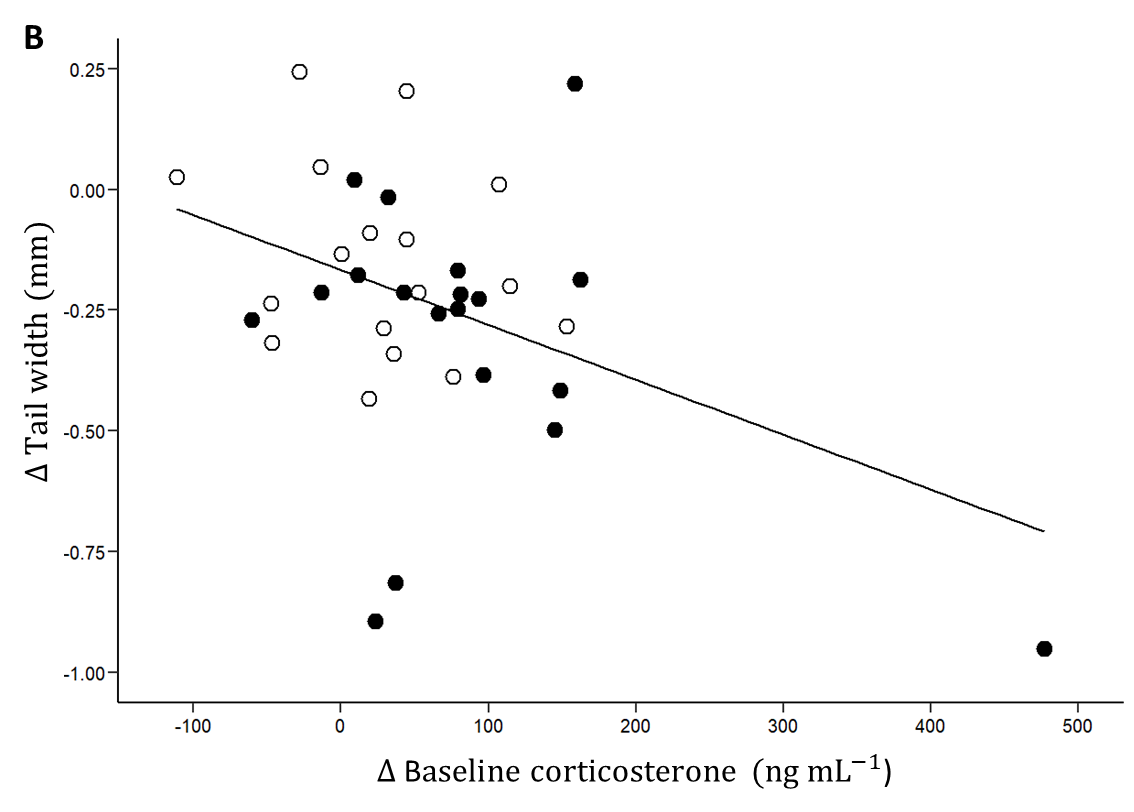

Supplement: Dezetter_et_al_Supplementary_File_coab071 [file dezetter_et_al_supplementary_file_coab071.docx]
